# CD133: a potential indicator for differentiation and prognosis of human cholangiocarcinoma

**DOI:** 10.1186/1471-2407-11-320

**Published:** 2011-07-29

**Authors:** Linni Fan, Furong He, Hongxiang Liu, Jin Zhu, Yixiong Liu, Zhiyong Yin, Lu Wang, Ying Guo, Zhe Wang, Qingguo Yan, Gaosheng Huang

**Affiliations:** 1 State Key Laboratory of Cancer Biology and Department of Pathology, Xijing Hospital, Fourth Military Medical University, Xi'an 710032, China; 2 Department of Histopathology, Addenbrooke's Hospital, Cambridge University Hospitals NHS Foundation Trust, Cambridge CB2 2QQ, UK; 3 Department of Cardiology, Xijing Hospital, Fourth Military Medical University, Xi'an 710032, China

**Keywords:** CD133, Cholangiocarcinoma, Immunohistochemistry, Differentiation, Prognosis

## Abstract

**Background:**

CD133 is known to be a cancer stem cell (CSC) marker. However, recent studies have revealed that CD133 is not restricted to CSC but to be expressed not only in human normal tissues but also in some cancers and could serve as a prognostic factor for the patients. Nevertheless, the expression of CD133 in human cholangiocarcinoma (CC) is rare and our study is to detect the expression and explore the potential functions of CD133 in human CC.

**Methods:**

Fifty-nine cases, comprised of 5 normal liver tissues and 54 consecutive CC specimens (21 well-differentiated, 12 moderately-differentiated and 21 poorly-differentiated), were included in the study. Immunohistochemical stainning with CD133 protein was carried out, and statistical analyses were performed.

**Results:**

CD133 was found to express in all 5 normal livers and 40 out of 54 (74%) CC tissues with different subcellular localization. In the well, moderately and poorly differentiated cases, the numbers of CD133 positive cases were 19 (19 of 21, 90%), 10 (10 of 12, 83%) and 11 (11 of 21, 52%) respectively. Further statistical analyses indicated that the expression and different subcellular localization of CD133 were significantly correlated with the differentiation status of tumors (*P = *0.004, *P *= 0.009). Among 23 patients followed up for survival, the median survival was 4 months for fourteen CD133 negative patients but 14 months for nine CD133 positive ones. In univariate survival analysis, CD133 negative expression correlated with poor prognosis while CD133 positive expression predicted a favorable outcome of CC patients (*P = *0.001).

**Conclusions:**

Our study demonstrates that CD133 expression correlates with the differentiation of CC and indicates that CD133 is a potential indicator for differentiation and prognosis of human CC.

## Background

CD133, also known as prominin-1, is a five-transmembrane domain molecule [1,2] located on apical plasma membrane protrusions of embryonic epithelial structures [3-5]. Up to now, it is mainly used for marking stem-like cells of various tissues and cancers [6]. Many tumors are known to contain a minority population of cancer stem cells or tumor-initiating cells which have the properties of self-renewal, proliferation, and multilineage differentiation and are responsible for sustaining the tumor [7]. Moreover, CD133 may represent a putative cancer stem cell marker in many solid tumors, such as human colon cancer [8,9], prostate tumor [10,11], pancreatic adenocarcinoma [12], renal cancer [13], neural tumor [14-17], and hepatocellular carcinoma [18,19].

It has been shown that CD133 is an apical molecule not only in embryonic epithelial structures, but also in many normal human tissues and its expression is not restricted to stem cells in pancreatic tissues [20]. In addition, CD133 has been found to be a prognostic factor of some cancers, such as colon and hepatocellular carcinoma [21,22]. Recently, Shimada et al. reported that normal bile duct epithelia were entirely negative for CD133 while 14 out of 29 CC cases expressed CD133. However, for each case, only a few tumor cells expressed CD133 protein; moreover, the 5-year survival rate in the CD133-positive group was worse than that in the CD133-negative group [23]. On the contrary, in our study we detected the expression of CD133 in human CC specimens by immunohistochemistry and analyzed its correlation with clinical and histopathological features. We found that CD133 was expressed on apical membrane of normal bile ducts and apical membrane or/and in the cytoplasma of CC tissues. The positive expression of CD133 on tumor cells was significantly correlated with well or moderately differentiated CC cases and predicted a better prognosis for the patients.

## Methods

### Clinical samples

Paraffin-embedded liver tissues from 54 consecutive patients with CC were retrieved from the pathology archives of Xijing Hospital between 2003 and 2007. In addition, 5 normal liver tissues were collected from the donor livers. The CC patients consisted of 28 males and 26 females with a mean age of 59.4 years (range from 34 to78 years). And the tumor specimens were composed of 25 cases of intrahepatic CC (ICC) and 29 cases of hilar CC (HCC) (Table 1). The patients' charts were reviewed and those who had pre-operative radiation or chemotherapy, or were immunocompromised, were excluded. All the histological slides were re-examined by two pathologists to ensure correct diagnosis of CC and the degree of its differentiation was evaluated in each case. Histological grading of CC was assessed based on the World Health Organization Classification of Tumors [24]. Among the 54 cases, 21 were well-differentiated, 12 were moderately-differentiated and 21 were poorly-differentiated. For clinical staging, 18 cases were at stage I/II while the other 36 were at stage III/IV. Moreover, 23 patients were followed up for survival from the date of surgery to March 31, 2008, including only that cacer-specific death of CC, the details of which were listed in additional file 1. Specimen collection and study procedures were approved by the Ethics Committee of Xijing Hospital.

**Table 1:** The origin and differentiation degree of the 54 cases of CC

Origin Differentiation degree	ICC	HCC	Subtotal
Well-differentiated glandular carcinoma	7	14	21
Moderately-differentiated glandular carcinoma	5	7	12
Poorly glandular carcinoma	13	8	21
Subtotal	25	29	54

ICC: Intrahepatic cholangiocarcinoma; HCC: Hilar cholangiocarcinoma

### Immunohistochemistry

Paraffin sections of 4 *μ*m thickness were deparaffinized and treated with 3% (v/v) hydrogen peroxide to block endogenous peroxidase activity. Heat-induced antigen retrieval was performed in 0.01 M sodium citrate (pH 6.0), followed by incubation in 10% (v/v) bovine serum albumin (BSA; Sigma, St Louis, MO, USA) in PBS (Phosphate Buffered Saline) at room temperature for 10 minutes to block the non-specific antibody-binding sites. The sections were then incubated overnight at 4°C with the rabbit monoclonal antibody against the human CD133 proteins (1:100 dilution; clone C24B9, Cell Signaling Technology, Danvers, MA, USA). Later on, a standard rapid EnVision technique (REAL™ EnVision™ Detection System, Peroxidase/DAB+, Rabbit/Mouse, Code K5007, Dako, Denmark) was used to detect the protein conjugates and develop the color. Finally, the sections were visualized after counterstaining with hematoxylin. Serial sections of CC were run in parallel with the primary antibody replaced by PBS and rabbit IgG1 (AMS/Immunokontact, Abingdon, UK) as blank and negative controls. The whole sections were screened for CD133 expression and the cholangiocarcinoma cases were defined as CD133 positive if CD133 staining was detected in more than 10% of the entire tumor area [25]. The slides were viewed under 100 × magnifications to define subcellular localization of the protein in the tumor glands. The evaluation of CD133 staining was carried out by two pathologists who had no knowledge of clinicopathological features of the cases.

### Statistics

Statistical analyses were performed using Statistical Program for Social Sciences (SPSS) software (Version 17.0, SPSS Inc, Chicago, USA). A Spearman correlation was used to analyze the correlation between the expression of CD133 and the clinicopathological features. The *Kaplan-Meier *analysis was also used to estimate tumor-specific survival, and different groups were compared with the *log-rank *test. For multivariate analysis, the *COX *regression model was used [26]. All tests were two-sided. *P *< 0.05 was considered significant.

## Results

### CD133 Expresses on Normal Bile Duct Epithelia and Most of the CC Tumor Cells with Different Subcelluar Localization

**Figure 1:**
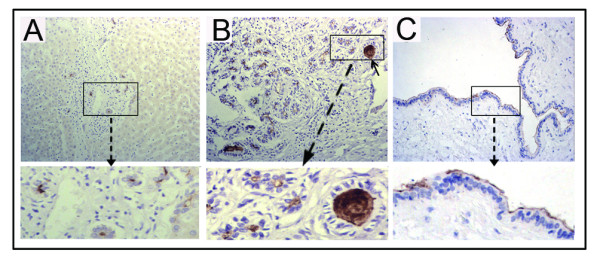
**Immunohistochemical staining of CD133 in normal human liver tissues**. CD133 expression was found mainly on the apical membrane surface of bile ducts of different sizes in normal liver tissue. (A) Normal small bile ducts, original magnification 200×; (B) Reactive bile ducts, original magnification 200×; (C) Large bile duct, original magnification 400×. In addition, CD133-positive cellular debris in the glandular lumina was observed in some normal bile ducts (B, arrow).

In the normal liver tissues, CD133 expression was found mainly on the apical membrane surface of bile ducts of all sizes (Figure 1A-C) but was not seen in hepatocytes, hepatic vessels and sinusoidal lining cells. In CC tissues, 40 of 54 cases (74%) were positive for CD133.

**Figure 2:**
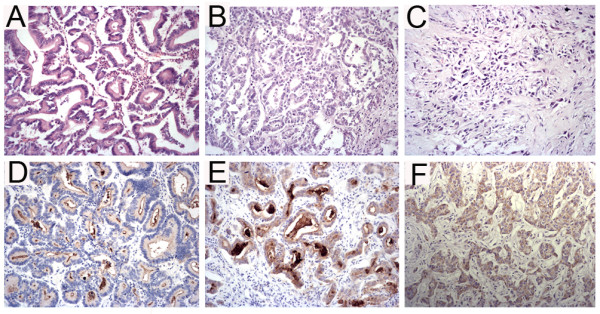
**The expression and different subcellular localization of CD133 in CC**. Among the CD133-positive cholangiocarcinoma, three staining patterns were identified: the well-differentiated tumors (A) were stained mostly on the apical membrane (D) or both membrane and cytoplasm (E) but the majority of moderately (B) and poorly differentiated (C) tumors were stained in the cytoplasm (F) or both membrane and cytoplasm, original magnification 400×. Also, in some cases, CD133-positive cellular debris was observed in the malignant glandular lumina (E).

The subcellular localization of the CD133 protein was investigated and three staining patterns, i.e. apical membrane, cytoplasm or both, were identified in CC (Figure 2D-F). In addition, some normal bile ducts (Figure 1B) and most CD133-positive tumors showed the shedding of CD133 positive cellular debris into the glandular lumina (Figure 2E).

### The Expression and Different Subcellular Localization of CD133 Correlate with the Differentiation Status of CC Tumors

In the well, moderately and poorly differentiated CC, the numbers of CD133 positive cases were 19 (19 of 21, 90%), 10 (10 of 12, 83%) and 11 (11 of 21, 52%) respectively. Spearman correlation test showed that the expression of CD133 was significantly correlated with well or moderately differentiated CC (*P *= 0.004, Table 2), which implied that the positive expression meant higher differentiation status of tumors while the absence of CD133 signified poorly differentiated ones.

**Table 2:** CD133 expression in CC

CC	Well differentiated	Moderately differentiated	Poorly differentiated	Subtotal
Number of cases	21	12	21	54
CD133 negative	2	2	10	14
CD133 positive	19 (90%)	10 (83%)	11 (52%)	40 (74%) *#
Membrane	8	2	1	11
Membrane & Cytoplasm	9	4	5	18
Cytoplasm	2	4	5	11

* *P *= 0.004 (CD133 positivity correlates with differentiation status);

# *P *= 0.009 (CD133 subcelluar localization correlates with differentiation status)

*P *< 0.05 is considered significant

Among the 40 cases of CD133-positive expression, the well-differentiated tumors were stained mostly on the membrane or on the both membrane and in the cytoplasm (17 of 19, 89%) but the majority of moderately and poorly differentiated tumors were stained in the cytoplasm or both on the membrane and in the cytoplasm (8/10, 80% and 10/11, 91%, respectively); moreover, by statistical anlysis, the different subcellular localization of CD133 correlated with the differentiation status of the tumor (*P = *0.009, Table 2). However, the age and gender of the patients did not correlate with the CD133 expression or the differentiation status of CC (*P >*0.05).

### CD133 Negative Expression in CC Predicts Worse Outcome

**Figure 3:**
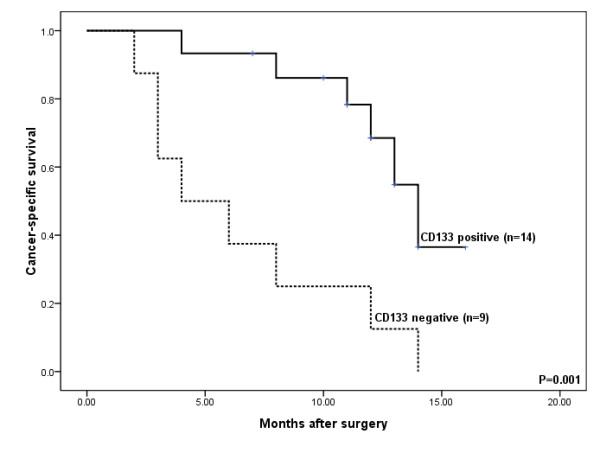
**CD133 negative expression correlates with poor survival in CC**. Kaplan-Meier survival curve showed the association between CD133 positive and negative expression in 23 patients with CC. Fourteen patients with positive CD133 expression had mean overall survival of 14 months versus 4 months for nine patients with CD133 negative expression. The log-rank test demonstrated CD133 negative expression correlated with poor outcome in CC (*P *= 0.001).

Twenty-three patients were followed up for survival. Among them, 14 cases were CD133 positive and the other 9 cases were CD133 negative in their tumors. Using Kaplan-Meier analysis, the median survival was 4 months for CD133 negative patients and 14 months for the positive ones. *Log-rank *test showed that the CD133 positive patients had a significantly better survival than negative ones (*P = *0.001) and the negative expression of CD133 was a worse prognostic indicator for patients with CC (Figure 3). The univariate analysis indicated that the differentiation status (*P *= 0.025), staging *(P = *0.006) and gender (*P *= 0.005) were also significant prognostic indicators, with the less well differentiated tumor and male gender predicating for poor overall survival for patients with CC (Table 3). To clarify the independent prognostic value of the CD133 expression in the patients with CC, a multivariate analysis of the relevant parameters was performed. The COX regression analysis revealed that CD133 expression (*P *= 0.007) and the differentiation status (*P *= 0.010) were prognostic factors for overall survival (Table 3).

**Table 3:** Prognostic variables for survival in 23 patients with CC

Variables	Number of cases	Median survival in months (range)	Univariate analysis (*P *value)	Multivariate analysis (*P *value)
Age				
< 60	14	14.00 (11.12-16.88)	0.058	0.086
> 60	9	11 (3.82-18.21)		
Gender				
Male	13	11 (7.15-14.85)	0.005	0.116
Female	10	17 (10.49-23.51)		
Differentiation status				
Well	8	15 (12.8-17.1)	0.025	0.010
Moderately	6	8 (2.24-13.7)		
Poorly	9	8 (0-19.7)		
Staging				
I/II	6	14 (12.5-15.5)	0.033	0.321
III/IV	17	11 (4.9-17.1)		
CD133 expression				
Positive	14	14 (11.9-16.1)	0.001	0.007
Negative	9	4 (0-8.2)		

*P *< 0.05 is considered significant

## Discussion

In this study, we investigated CD133 expression in normal human liver tissues and CC tissues using the immunohistochemical method. We found that CD133 was expressed in human normal livers but the expression was restricted to biliary trees on the apical membrane. In CC, 40 of 54 (74%) cases studied showed CD133 expression with different degrees. Similar to colon and mammary gland adenocarcinoma which have high expression of CD133 in well or moderately differentiated tumors [27], most of the well or moderately differentiated CC (90% and 83%, respectively) expressed CD133 and moreover, most CD133 negative CC tumors (71%) were poorly differentiated.

Our study and other researches have found that CD133 expression pattern varies in different cancers. In colorectal carcinoma, CD133 positivity is restricted only to the luminal surface [21,28]. In our study, besides the luminal surface pattern in most well-differentiated CC cases, most of moderately or poorly differentiated CC cases showed cytoplasmic or both cytoplasmic and membranous staining (Table 2). It is believed that the different subcellular localization of CD133 (cytoplasm or membrane) may confer different cellular functions [20]. The protein is expressed apically on the membrane of epithelial cells when a lumen has been formed but it is expressed in the cytoplasm of solidly arranged malignant tissues of non-epithelial origin [20]. It is well known that if a formed lumen is observed in an epithelial tumor, it usually indicates for better differentiation of the glandular tumor. In our study, CD133 showed cytoplasmic expression in many CC cases, suggesting that cytoplasmic CD133 expression should be not just restricted to malignant tissues of non-epithelial origin [20] but also occurs in malignant epithelial cells of CC. The statistical analysis showed that CD133 expression significantly correlated with the differentiation status of CC (*P = *0.004). It is possible that the expression of CD133 on the apical membrane may indicate a particular stage of the cell differentiation connected with the formation of lumen and ducts as same as the normal bile ducts, and this deduction is confirmed by Shmelkov, S.V. et al in their study [28]. In addition, we noticed the CD133 positive debris in normal bile ducts and the shedding of CD133 positive tumor cells and apparent non-cellular, CD133-positive materials in the malignant ducts. This observation is in accordance with the previous report that small membrane vesicles containing prominin-1 were found in the human normal saliva and tears as well as malignant lumen of colorectal cancer [29,30]. The concentration of CD133, presented within the apical membrane of glands or in extracellular membranous vesicles, suggests that this cholesterol-binding glycoprotein might play a role in regulating the secreting process [29,31]. Similarly, CD133 expression in normal bile ducts and its immunoreactivity associated with the secretion therein indicate that CD133 protein might exist in bile, thus, a quantitive analysis of CD133 in bile might be helpful for distinguishing CC from hepatocellular carcinoma.

Many previous researches have demonstrated the importance of CD133 as a defining factor of the cancer stem cell phenotype, including human liver cell line [19] and hepatocellular carcinoma [32]. In this study, however, we found that CD133 was commonly expressed on the apical membrane of all normal bile ducts and was frequently expressed in the majority of CC cases, which were clearly at the differentiation stages beyond the stem/progenitor cells. Other studies also find that the overall expression of human CD133 is beyond the rare primitive cells and appears to be a general marker for apical membrane of fully differentiated glandular epithelia [4,20,28,33]. These findings suggest that it should be inappropriate to employ CD133 as morphological characterization of cancer stem cells and the value of CD133 expression as a marker for cancer stem cells should be critically evaluated in future studies [34].

Recently, several studies have reported that the presence of CD133 in various tumors is correlated with poor prognosis. Zeppernick [35] and Beier [17] have found that CD133 expression indicates shorter survival for adverse gliomas and high-grade oligodendroglial tumors. Similarly, the high expression of CD133 in colorectal adenocarcinoma is related to poor survival of the patients [21,25]. Furthermore, the high expression of CD133 is correlated with the increased tumor grade, advanced disease stage, elevated serum alpha-fetoprotein levels and poor survival of the patients with hepatocellular carcinoma [21]. In this study, we found that CD133 expression is also significantly associated with the survival of the patients with CC. However, there are differences between our findings and the previous reports. Firstly, the patients of CC with positive CD133 expression had significantly better prognosis than those negative ones. Secondly, the patterns of CD133 expression varied in different human CC cases and seemed to be related to differentiation stages of the tumors. Like glandular epithelia that usually show apical membrane expression pattern of CD133 [20,33], normal bile ducts showed the same expression pattern. Interestingly, similar expression pattern of CD133 was found in well-differentiated CC. In contrast, poorly differentiated CC cases usually showed solid growth pattern and most of them were cytoplasmic positive or even negative for CD133. Nevertheless, in the patients with pancreatic ductal adenocarcinoma, there is no correlation between the level of CD133 expression and patient survival [20]. However, Maeda S et al found that the 5-year survival rate of CD133-positive patients was significantly lower than that of CD133-negative patients [36].

Therefore, the role of CD133 expression in evaluating prognosis of cancer patients needs to be explored in further work. Until now, the physiological role of CD133 is still elusive probably due to the occurrence of many mRNA splice variants and the changing glycosylation status of the protein [37,38]. Moreover, the protein has different subcelluar localizations (membrane, cytoplasm or both), which may confer different functions. It also remains to investigate why CD133 is usually present on apical membrane of normal bile ducts or other gland lumen, why it is transferred from apical membrane to cytoplasm in cancer cells and what its biological functions in normal bile duct epithelium and cholangiocacinoma are. And the answers to these questions could help us to understand the role of CD133 in the development of CC and other tumors.

## Conclusions

We found CD133 expressed mainly on the apical membrane surface of bile ducts of all sizes and most of malignant ducts. And the subcellular localization of the CD133 protein was investigated and three staining patterns, i.e. apical membrane, cytoplasm or both, were presented in CC and the first pattern was frequently found in well-differentiated CC. In addition, the positive expression of CD133 was significantly correlated with well or moderately differentiated CC and vice versa. Moreover, the patients of CC with positive CD133 expression had a significantly better prognosis than those negative ones. In brief, our study demonstrates that CD133 expression correlates with the differentiation of CC and indicates that CD133 is a potential indicator for differentiation and prognosis of human CC, the mechanisms of which, however, need to be investigated in further study.

## Abbreviations

CSC: cancer stem cell; CC: cholangiocarcinoma; ICC: intrahepatic cholangiocarcinoma; HCC: hilar cholangiocarcinoma

## Competing interests

The authors declare that they have no competing interests.

## Authors' contributions

LF performed the statistical analysis and drafted the manuscript. FH carried out immunohistochemistry staining and drafted the manuscript. HL helped to revise the draft. JZ and YL participated in collecting the samples. ZY carried out the figures and tables. LW and YG participated in the follow-up study. ZW and QY helped to confirm the diagnosis of the samples. GH conceived the study, and participated in its design and coordination and helped to draft the manuscript. All authors read and approved the final manuscript.

## Pre-publication history

The pre-publication history for this paper can be accessed here:

http://www.biomedcentral.com/1471-2407/11/320/prepub

## Supplementary Material

Additional file 1 **The differentiation grade and CD133 expression of 23 follow-up cases**. The table contained the details of differentiation grade and CD133 expression of all 23 follow-up cases. The differentiation grade was classified as well-, moderately- and poorly-differentiated, and the CD133 expression was presented with "+"as positive while "-" as negative. Click here for file
