# Supplementary material for: CD133: a potential indicator for differentiation and prognosis of human cholangiocarcinoma
Source: BMC Cancer. 2011 Jul 29;11:320. doi: 10.1186/1471-2407-11-320 (PMC3161038; doi:10.1186/1471-2407-11-320)
Supplement: Additional file 1 — The differentiation grade and CD133 expression of 23 follow-up cases. The table contained the details of differentiation grade and CD133 expression of all 23 follow-up cases. The differentiation grade was classified as well-, moderately- and poorly-differentiated, and the CD133 expression was presented with "+"as positive while "-" as negative. [file 1471-2407-11-320-S1.DOC]

**Additional file 1**

**The differentiation grade and CD133 expression of 23 follow-up cases**

| Cases No. | Differentiation grade | CD133 expression |
| --- | --- | --- |
| 1 | P | - |
| 2 | P | - |
| 3 | P | + |
| 4 | W | - |
| 5 | W | + |
| 6 | P | - |
| 7 | W | + |
| 8 | M | - |
| 9 | P | + |
| 10 | M | + |
| 11 | P | + |
| 12 | W | + |
| 13 | M | + |
| 14 | M | + |
| 15 | W | + |
| 16 | M | - |
| 17 | P | + |
| 18 | P | - |
| 19 | W | + |
| 20 | M | + |
| 21 | W | + |
| 22 | W | + |
| 23 | P | - |

W: well-differentiated; M: moderately-differentiated; P: poorly-differentiated;

+: positive; -: negative
